# DEMENTIA KNOWLEDGE IN CHINESE NEWSPAPERS (2005–2020): A TOPIC MODELING ANALYSIS

**DOI:** 10.1093/geroni/igae098.0192

**Published:** 2024-12-31

**Authors:** Lin Chen, Felicia F Tian, Yu Fu

**Affiliations:** Fudan University, Somerville, Massachusetts, United States; Fudan University, Shanghai, Shanghai, China (People’s Republic); Fudan University, Shanghai, Shanghai, China (People’s Republic)

## Abstract

How public policies convey dementia is an important source of the public’s understanding of dementia, and newspapers are critical to depicting and disseminating this information to the public. The present study used topic modeling strategies to analyze Chinese newspaper portrayals of dementia from 2005 to 2020 to trace changes in key areas of dementia knowledge in relevant policies. Using WiseNews, the largest Chinese media database, we chose 45 newspapers from mainland China and identified 12,719 articles related to dementia. Using latent Dirichlet allocation (LDA), we performed a topic modeling analysis and identified the six most prevalent topics on dementia across articles: lifestyle recommendations, neighborhood life, foundational scientific research, celebrity and media portrayals, dementia caregiving, and pharmaceutical innovations—all related to the dementia knowledge scale’s four dimensions. Findings suggest a steady increase in the number of articles on dementia caregiving and a decline in lifestyle recommendations from 2005 to 2020. However, newspapers continued to stigmatize aging by regularly co-depicting dementia and old age and by using biased terminology. Among the first to investigate dementia’s portrayals in mainland Chinese newspapers, this study illuminates the need for expanding mass media campaigns to raise the country’s dementia knowledge to foster a dementia-inclusive society.

